# Rhythmic Changes in Synapse Numbers in *Drosophila melanogaster* Motor Terminals

**DOI:** 10.1371/journal.pone.0067161

**Published:** 2013-06-28

**Authors:** Santiago Ruiz, Maria Jose Ferreiro, Kerstin I. Menhert, Gabriela Casanova, Alvaro Olivera, Rafael Cantera

**Affiliations:** 1 Departamento de Biología del Neurodesarrollo, Instituto de Investigaciones Biológicas Clemente; Estable, Montevideo, Uruguay; 2 Unidad de Microscopía Electrónica de Transmisión, Facultad de Ciencias, UdelaR, Montevideo, Uruguay; 3 Zoology Department, Stockholm University, Stockholm, Sweden; University of Edinburgh, United Kingdom

## Abstract

Previous studies have shown that the morphology of the neuromuscular junction of the flight motor neuron MN5 in *Drosophila melanogaster* undergoes daily rhythmical changes, with smaller synaptic boutons during the night, when the fly is resting, than during the day, when the fly is active. With electron microscopy and laser confocal microscopy, we searched for a rhythmic change in synapse numbers in this neuron, both under light:darkness (LD) cycles and constant darkness (DD). We expected the number of synapses to increase during the morning, when the fly has an intense phase of locomotion activity under LD and DD. Surprisingly, only our DD data were consistent with this hypothesis. In LD, we found more synapses at midnight than at midday. We propose that under LD conditions, there is a daily rhythm of formation of new synapses in the dark phase, when the fly is resting, and disassembly over the light phase, when the fly is active. Several parameters appeared to be light dependent, since they were affected differently under LD or DD. The great majority of boutons containing synapses had only one and very few had either two or more, with a 70∶25∶5 ratio (one, two and three or more synapses) in LD and 75∶20∶5 in DD. Given the maintenance of this proportion even when both bouton and synapse numbers changed with time, we suggest that there is a homeostatic mechanism regulating synapse distribution among MN5 boutons.

## Introduction

Neurons change morphology following circadian rhythms, which are influenced by light, glial cells, neurotransmitters and proteins encoded by “clock genes”, among other factors. This special type of neuronal plasticity has been vastly documented through the study of several types of fly neurons (reviewed in [1], [2], [3]) and has also been demonstrated in several species of vertebrates [4]–[7].

“Synaptic boutons” is the term used to define discrete swellings of the axonal terminal in contact with the target muscle, within which synapses are localized. In larval axons of the fly *Drosophila melanogaster*, synaptic boutons are dynamic structures that can appear, grow, subdivide or disappear in a few hours [8], [9]. In the neuron studied here, *Drosophila* flight motor neuron 5 (MN5) of the adult *Drosophila*, synaptic boutons increase in diameter during the day and shrink again during the night following a circadian rhythm, which is not present in flies with mutations in the clock genes *period* and *timeless* or in old wild type flies [10]. Given that the term “synapse” is used in the scientific literature to describe different structures (a semantic issue discussed by Collins and DiAntonio [11]), we would like first to specify that we use the term “synapse” as synonymous for “active site”, identified with electron microscopy as a place where presynaptic and postsynaptic membranes are more electron dense and parallel to each other, with a cluster of synaptic vesicles and often a presynaptic ribbon termed “T-bar” on the presynaptic side [11]–[15]. The proportion of synapses without T-bars ranges from 15 to 25% depending on the type of motor neuron and fly stock [16]–[18]. A single bouton might lack synapses entirely (“empty bouton”) or contain a combination of synapses of different age. In *Drosophila*, synapses between photoreceptors and visual interneurons are formed within minutes [19] but neuromuscular synapses take most probably a few hours to be formed or dismantled [12], [20], [21].

The biological relevance of circadian rhythms in neuronal shape is not well understood. In the case of motor neurons, the rhythmic change in bouton size could be related to the rhythmic pattern of locomotion activity, which in *Drosophila* comprises alternating intervals of activity and rest [22] with a prolonged period of sleep/rest during the night [23], [24]. However, an experimental approach to test this hypothesis indicated that the rhythm in bouton size was largely independent of synaptic activity [25]. Circadian changes in membrane excitability have been reported for a subset of “clock neurons” [26], [27] but electrophysiological studies of the activity of motor neurons at different times of the day are still not available. On a purely speculative basis, it has been proposed that a nocturnal reduction in the size of motor terminals could provide a less energetically demanding morphology during the night [10] while the fly is resting [23], [24], [28]. This could have adaptive value because it will reduce the high metabolism associated with axonal transport and other biological processes demanded for the maintenance of motor synapses during a substantial part of the fly’s life [10]. From this point of view, consistent with the “synaptic homeostasis” hypothesis of Tononi and Cirelli [29], it is plausible to assume that the nocturnal reduction of bouton size in flight motor neurons also includes a reduction of synapse numbers. However, data on synapse numbers in motor neurons at different times of the day is still not available for neurons from *Drosophila* or other animal species.

Our knowledge on circadian changes in synapse numbers is restricted to a few neuronal types from a few animal species. The scarcity of the data and the variability of results preclude any sound generalization and further elaboration of explanatory models. Some neurons keep synapse numbers constant over several days while others have more synapses during the night or during the day (see below). In some cases the results differ when the experimental animals are kept in constant darkness, a condition that does not radically change their circadian rhythm of activity/rest. Moreover, there are examples of neurons of nocturnal animals with more synapses during the day and of neurons of diurnal animals with more synapses during the night. In the housefly (a diurnal animal), under light:darkness (LD) cycles, two types of synapses increase in number once a day with opposite phase. Photoreceptor synapses on the visual interneuron L2 (“tetrad synapses”) are more abundant during the day and L2 synapses on photoreceptors (“feedback synapses”) are more abundant during the night. Only the “feedback synapses” exhibit the rhythm when flies are kept in constant darkness (DD) [30], [31]. In the fruit fly (also diurnal), the synapses formed by retinal axons onto visual interneurons L1 and L2 increase in number during the day [32]. In the rat (a nocturnal animal), careful counting of synapses in the suprachiasmatic nucleus of animals kept in different conditions gave different results for different synapse types. When comparing rats kept in constant light or darkness, ultrastructural changes were detected in synaptic boutons and synapses of optic afferents but without changes in synapse numbers [33]. In rats kept in LD, axo-somatic synapses made by glutamatergic and non-glutamatergic neurons onto vasoactive intestinal peptide producing neurons increased by 36% at daytime, whereas no changes were detected in the number of synapses made on arginin-vasopressin expressing neurons [6]. A similar finding regarding axo-dendritic synapses on vasoactive intestinal peptide producing neurons was that the number of GABAergic synapses did not change but that of non-GABAergic synapses increased by 62% during daytime [7]. In spite of these selective changes, the authors proposed that the global number of synapses in the suprachiasmatic nucleus remains constant between day and night [6], [7]. Finally, in the zebrafish (a diurnal animal), hypocretin/orexin neurons projecting to either the hindbrain or pineal gland show a rhythmic change in the number of synapses in LD, with more synapses during the transition between light and darkness [5]. This rhythm persists under DD and exhibits different phases according to the brain area where those synapses were counted (hindbrain or pineal gland).

In the present work, we use two complementary microscopy methods (Transmission Electron Microscopy and Laser Confocal Microscopy) to count boutons and synapses in a motor neuron of *Drosophila melanogaster* at different time points of the day to investigate the possibility of a daily rhythm in synapse numbers. One of the many advantages of using *Drosophila* motor neurons for this type of studies is that good estimations of synapse numbers can be obtained in relatively short time without the aid of the electron microscope, a method that has the advantage of giving higher resolution but that is exceedingly time demanding. The most widely used method to count synapses in the *Drosophila* neuromuscular junction is to stain this structure with specific fluorescent markers (i.e. antibodies against Horseradish peroxidase to outline the neuronal membrane [34] and antibodies against synaptic proteins), to study the preparation with laser confocal microscopy and to count the immunofluorescent “spots” (each representing a synapse). A monoclonal antibody (nc82, [35]) specific for the synaptic protein Bruchpilot (BRP, an ELK/CAST homologue, [15]) has became widely used to stain *Drosophila* neuromuscular synapses because of its many advantages [12], [15], [21], [36]–[46]. On one hand, antibody nc82 gives very consistent results with beautifully distinct, strongly fluorescent “spots” against a practically negative background, making very easy to count the stained structures [12], [21], [37], [39]–[42], [47]–[49]. On the other hand, a wealth of experimental cell biology, genetics and electrophysiology supports the idea that the great majority of “nc82 spots” do indeed represent individual synapses along the neuromuscular junction. When the BRP immunostaining at the neuromuscular junction was observed with the electron microscope, it appeared exclusively at synapses [12]. Double staining for nc82 and antibodies specific for glutamate receptors defined that practically each BRP-spot (presynaptic side of a single synapse) correlated with a single cluster of glutamate receptors (postsynaptic side of the same synapse) reinforcing the view that anti-BRP alone can be used to obtain a good estimation of synapse numbers [12], [15], [36], [38], [39], [43], [46]–[48], [50].

## Materials and Methods

### Flies and Rearing Conditions

Wild-type flies of the strain Oregon R were raised on standard *Drosophila* medium at 25°C and kept at LD cycles of 12∶12 hours with Zeitgeber time 0 (ZT0; lights-on) set at 09∶00 am. For DD experiments, flies were kept in LD cycles for at least three days before the light was switched off at ZT0 the first day of the experiment (setting the circadian time 0, CT0 in DD). All experiments were done with 4–5-days old female flies because they are known to exhibit strong rhythmicity in bouton size [10], [23]. The same two time points were studied in both conditions of illumination: ZT19 (midnight, in the middle of the phase of sleep), and ZT7 (midday) in LD cycles, and CT19 (subjective midnight) and CT7 (subjective midday) in DD. The flies were anesthetized with nitric oxide (Sleeper TAS, INJECT+MATIC, Switzerland), decapitated with a sharp needle and thereafter the dorsal portion of the thorax was dissected and fixed for either method as described below (TEM: in LD, n = 7 flies per ZT and in DD, n = 6 per CT; LCM: in LD, ZT19 n = 38 flies and ZT7 n = 43; in DD, CT19 n = 33 and CT7 n = 37, Table S1 and Table S2).

### Transmission Electron Microscopy (TEM)

The samples were prepared for TEM according to the method detailed in [51]. The neuromuscular junction formed by the MN5 is extraordinarily large and complex compared to other motorneurons. This precludes the possibility of counting every single bouton or generating as many complete reconstructions of them as it will be necessary to obtain a representative sample of the thousands of boutons involved in a single terminal. The method adopted here instead, is based on the analysis of single sections complemented with a few serial reconstructions of single boutons. Every section contained the two longitudinal indirect flight muscles (IFM5 and IFM6) innervated by MN5 [52], [53]. Two time points (ZT19/CT19 or “midnight” and ZT7/CT7, or “midday”) throughout two consecutive LD or DD cycles were analyzed. Then, after it was observed that the results were very similar for both consecutive cycles, data from the same time points were pooled in each condition of illumination. A minimum of 80 images per time point were analyzed (LD, 260 boutons in ZT19 and 168 boutons in ZT7; DD, 215 boutons in CT19 and 184 boutons in CT7). The ultrathin sections (40 to 60 nm) were observed with a JEOL JEM 1010 operated at 80 kV and the images were taken with a digital camera (Hamamatsu C4742-95) at magnification 40,000×, 100,000× or 150,000×. Image processing and counts were done with AMT Advantage CCD, Adobe Photoshop and Photoimpact softwares.

### Laser Confocal Microscopy (LCM)

A double staining was performed to count boutons and synapses. The MN5 neuronal membrane was outlined by immunostaining with a rabbit polyclonal anti-Horseradish Peroxidase serum (Jackson ImmunoResearch), which stains insect neuronal membranes [34] and specifically recognizes the glycoprotein 3-alpha-L-fucosyltransferase [54]. Simultaneously, synapses were marked with monoclonal antibody nc82 (DSHB) that specifically recognizes the protein Bruchpilot, a well documented marker of synapses [12], [15], [21], [36]–[50]. The antibody recognizes a region of the synapse [12], [55]. Additionally, a triple staining was performed to confirm the presence of synaptic vesicles in the presynaptic side of the synapse and glutamate receptors in the postsynaptic side. Synaptic vesicles were marked with monoclonal antibody 3C11 (DSHB) that specifically recognizes the protein Synorf1 [56], [57] and glutamate receptors were marked with monoclonal antibody 8B4D2 (DSHB) that specifically recognizes the glutamate receptor subunit IIA [58]. The dorsal half of the thorax was dissected in a droplet of phosphate-buffered saline (PBS, 0.1 M at pH 7.4), bisecting the thorax along the midsagittal plane to improve antibody penetration. Only one hemithorax per fly was processed further. The samples were fixed in ice-cold 4% paraformaldehyde buffered at pH 7.4 for 2.5 hours or in Bouin’s solution (for 8B4D2 antibody) for 30 minutes. Tissues were then permeabilized in 0.3% Triton X100 in PBS (PBST) and unspecific binding sites were blocked with 1% Normal Goat Serum in PBST with 0.5% bovine serum albumin (PBSTA). The samples were then incubated with the primary antibodies (rabbit anti-HRP diluted 1∶600, mouse nc82 diluted 1∶100, 3C11 and 8B4D2 both diluted 1∶10) over-night at room temperature (RT). The next day, the samples were washed 4×15 min in PBST before being incubated with the corresponding secondary antibodies for 2 hours at RT (goat anti-rabbit conjugated to Alexa 488 from Molecular Probes, diluted 1∶1000 in PBST and goat anti-mouse conjugated to Cy3 from Jackson ImmunoReseach, Inc., diluted 1∶1000). In triple staining, given that two of three antibodies used were monoclonal antibodies of the same species, samples were incubated with the corresponding combination of secondary isotype-specific antibodies (goat anti-mouse IgG2b Alexa 568 from Molecular Probes, diluted 1∶1000 in PBST and goat anti-mouse IgG1 Alexa 488 from Molecular Probes, diluted 1∶1000). The samples were rinsed 4×15 min in PBS and thereafter IFM5 and IFM6 were dissected in PBS with a sharp needle and mounted in 80% glycerol. All samples were examined with a laser confocal microscope Olympus FV300. Each final image was a projection of a Z-series of approximately 15 optical sections scanned at intervals of 0.3 µm with a 60× lens and a digital zoom of 3.5, covering the whole thickness of the muscle under study. Two such images were done for each fly. The quality of the colors was optimized with ImageJ software and the number of boutons and synapses was counted in each image working “blind”. For the analysis of synapse profiles, the fluorescence intensity was measured with ImageJ software using the plug-in Colour Profiler.

### Quantification of Boutons and Synapses

**Figure 1:**
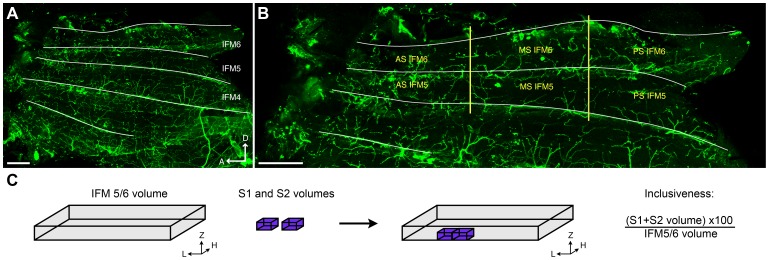
Inclusiveness of the LCM sampling method used for the study of the MN5 terminals. (A) The figure shows a panoramic view of an hemithorax showing muscles IFM4, IFM5 and IFM6 (from ventral to dorsal: dorsal, D and anterior, A). The bar scale represents 100 µm. (B) Magnification of the same hemithorax presented in A, with yellow lines showing the regions of flight muscles IFM5 and IFM6 considered for the analyses (anterior stack, AS; medial stack, MS; posterior stack, PS). The bar scale represents 100 µm. (C) Schematic representation showing how the inclusiveness was calculated. The total volume of muscles IFM5 and IFM6 was calculated measuring thickness, height and sagittal length of each muscle (in four flies). These measurements were done in MS IFM6 and MS IFM5 (see panel B). The volume for the stacks scanned in each sample (fly) (S1 and S2 volumes) was calculated knowing that each stack comprised approximately 15 optical sections scanned at intervals of 0.3 µm with a 60× lens and a digital zoom of 3.5. Inclusiveness was calculated as the sum of S1 and S2 volumes divided by the total muscles IFM5/6 volume.

Two microscopy methods (TEM and LCM) were used to count boutons and synapses at each ZT or CT. As the counting of all boutons and synapses in the terminal of the MN5 over and inside its target muscles IFM5 and IFM6 is practically not feasible given its neuroanatomy and size, we counted those features in two random samples per fly, each representing a relatively small volume of the entire MN5 terminal. We evaluated the inclusiveness and reliability of this method. The inclusiveness of the samples of the muscle analyzed (two stacks per fly) was calculated as the volume of the stacks divided by the total volume of the muscles and expressed as a percentage. The volume of the stacks was calculated using their thickness, height and sagittal length (approximately 15 optical sections scanned at intervals of 0.3 µm with a 60× lens and a digital zoom of 3.5) (Fig. 1). The total volume of the muscles IFM5 and IFM6 was calculated using the mean of the thickness, height and sagittal length of these muscles. This was measured in four flies. To evaluate the reliability of our sampling method we tested if there were significant differences in the counts depending on where in the muscle the stacks were done. This was evaluated in four flies, taking three confocal stacks per fly (ZT19) each in one of three different regions of the IFM5 and IFM6 muscles (anterior, medial and posterior region; Fig. 1B, Table S3), counting boutons, synapses, boutons with and without synapses, and investigating if there were statistical differences between regions. The proportion of boutons per time point was calculated as the number of boutons found in each time point divided by the number of boutons found in the experiment. The same calculation was done for the proportion of boutons with synapses. For TEM, the proportion of boutons with synapses was quantified as the number of boutons with synapses found in each time point divided by the number of boutons with synapses found in the experiment. Finally, the proportion of boutons with and without synapses per time point (relative to the total number of boutons found in each time point) and synapse distribution among boutons was analyzed in LCM. Boutons with synapses were classified into three classes: boutons with one, two, or more synapses and the percentage of each subpopulation were calculated. Thereafter the mean ± s.e.m was calculated and graphically represented.

### Statistical Analysis

Student *t*-tests were performed when assumptions for parametric test were accomplished (normality using Shapiro-Wilk W test and homoscedasticity using Levenés test). If these assumptions were not achieved, nonparametric Kruskal -Wallis and Mann-Whitney U tests were performed instead. Statistical significance was set at 0.05, 0.01 and 0.001. Analyses were done using Statistica 7.0 software (Statsoft).

## Results

### General Neuroanatomy of the MN5 Axonal Arborization

**Figure 2:**
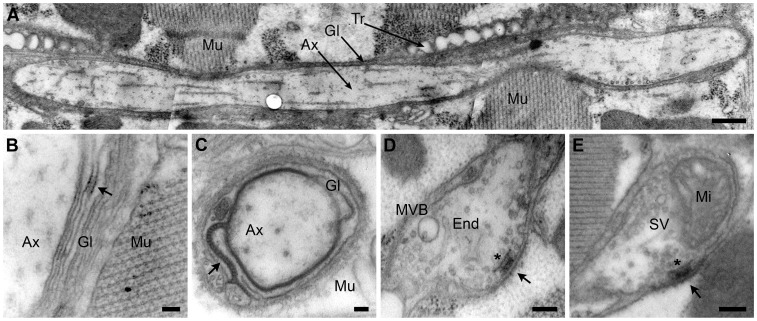
Neuroanatomy of the neuromuscular junction of motor neuron 5 (MN5) studied with TEM. (A) The adult MN5 forms many axonal branches (Ax) that penetrate deeply into longitudinal flight muscles (Mu) covered by glia (Gl) and accompanied by tracheae (Tr). (B) When the primary branches of the MN5 axon (Ax) reach their target muscle (Mu), they are covered by up to 10 concentric layers of glia (Gl) forming septate junctions (only a small portion of such junction is seen here, arrow). (C) Inside the muscle (Mu), secondary axonal branches (Ax) are covered by a single glial layer (Gl) with a meandering septate junction (arrow). Thickenings of the axonal branches (synaptic boutons, D and E) form synapses (black arrows in D and E) and contain mitochondria (Mi in E), endosomes (End in D), multivesicular body (MVB in D) and other organelles. Some synapses contain a presynaptic density called T-bar (asterisk in D and E). There is a large variation among boutons in the number of synaptic vesicles (SV; compare D with E, see also [51]). Scale bars: 1 µm (A), 100 nm (B), 200 nm (C), 500 nm (D) and 100 nm (E).

The neuroanatomy of MN5 has been studied with different methods [10], [53], [59]-[62]. A brief description of the major differences between this neuron and the majority of larval (reviewed in [63]) and adult [64]-[66] *Drosophila* motor neurons studied so far is given below. One major difference is that the adult MN5 axon makes hundreds of branches [10], [52], [53], an order of magnitude more than all other *Drosophila* motor neurons. Furthermore, contrary to what is the rule for larval and most adult motor terminals, we found that the axonal branches of the MN5 penetrated both target muscles and did so in tight association with glia (Fig. 2A). The primary axonal branches on the surface of the muscle were wrapped by a glial cell forming up to ten concentric layers (Fig. 2B) with a septate junction. Secondary and tertiary axonal branches penetrated the muscle and were also wrapped by glia, although in this case only with a single layer and a septate junction (Fig. 2C). The synaptic boutons were not covered by a subsynaptic reticulum (Fig. 2D, E). About 90% of the images of adult MN5 boutons containing synapses had only one synapse instead of several as is seen frequently in images of larval boutons [11], [63], [67]. Many of our bouton images showed very few vesicles in accordance with previous studies [51], [68]. This stands in contrast with larval motor terminals, in which practically every ultra-thin section across a synaptic bouton contains abundant synaptic vesicles [11], [63], [67]. Furthermore, we found that the majority of MN5 boutons were located inside the muscle (i.e. completely surrounded by muscle cytoplasm) whereas in larval muscles practically all boutons are located on the surface of the muscle.

### The Numbers of Boutons and Boutons Containing Synapses are Increased at Midnight

Our analyses of inclusiveness and reliability showed that the microscopy and sampling methods used here generated representative and reproducible results. Each couple of confocal stacks used to count boutons and synapses in each fly represented about 5% of the total volume of the corresponding IFM5 and IFM6 muscles (Fig.1 and Table 1). We found no statistical differences between results obtained when these two stacks were scanned in different regions of the muscles, indicating that boutons and synapses are homogeneously distributed across these muscles (Fig. 1 and Table 1). Using these data we estimated that a single MN5 has approximately 4000 boutons and 3000 synapses.

**Table 1:** Measurements for the analysis of Inclusiveness and Reliability.

	Thickness (Z; µm)	Height (H; µm)	Sagittal length (L; µm)	Calculated sagittalarea (µm^2^)	Volume (calculated sagittal area)(µm^3^)
Muscle 6	41.1	114.3	533.2	60944.8	2504829.6
	41.1	87.1	629.8	54855.6	2254564.3
	44.4	94.1	718	67563.8	2999832.7
	38.1	82.8	708.4	58655.5	2234775.3
Mean	41.2±1.3	94.6±7	647.4±42.9	60504.9±2667.0	2498500.5±178051.9
Muscle 5	34.2	121	555.4	67203.4	2298356.3
	42.9	109.2	759	82882.8	3555672.1
	50.7	73.9	729.7	53924.8	2733988.9
	35.1	60	867.4	52044.0	1826744.4
Mean	40.7±3.9	91.0±14.4	727.9±64.7	64013.8±7137.2	2603690.4±367437.2
	**Height (µm)**	**Area (µm^2^)**	**Thickness (min-max;** **mean; µm)**	**Mean Volume (Stack 1** **or Stack 2; µm^3^)**	**Two images per fly (Stacks 1+2; µm^3^)**
Stack (60×, x3.5)	67.67	4579.2	11.7–14.4; 13.0±1.3	59758.9	119517.9
	**Inclusiveness (%)**				
Muscle 6	4.8				
Muscle 5	4.6				

**Figure 3:**
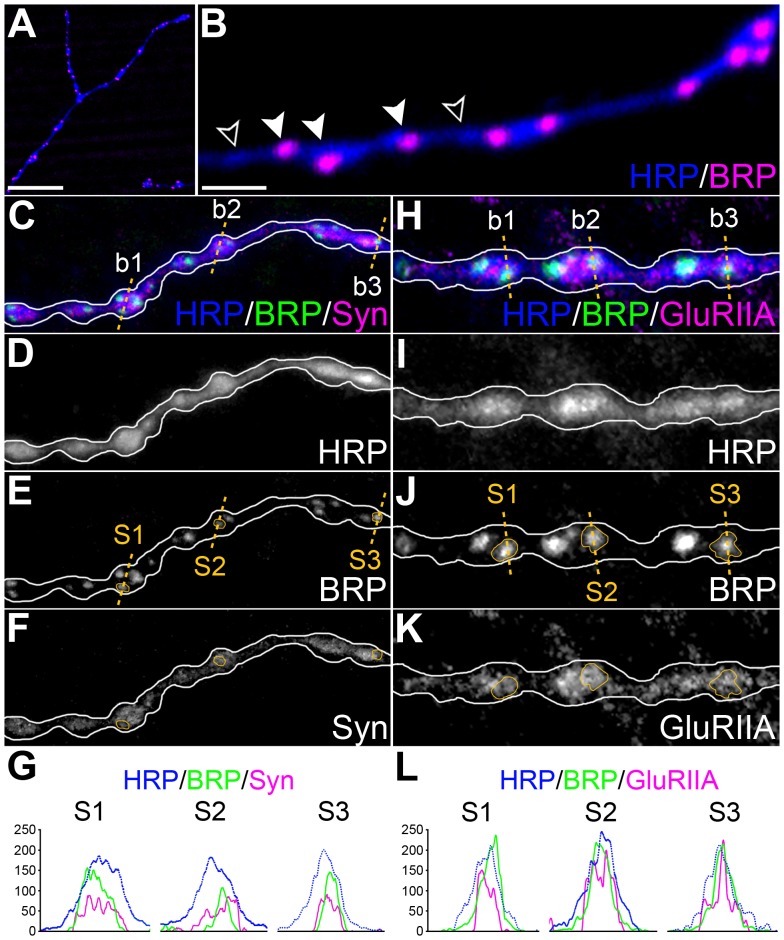
Visualization of synapses in the MN5 terminal based on synaptic markers and LCM. (A) Example of the staining of the MN5 neuromuscular junction with anti-Horseradish Peroxidase (HRP, blue) and anti-nc82 (BRP, magenta) in some of the thinnest branches, located deeply inside the muscle. The bar scale represents 10 µm. (B) Example of the same staining in a branch of this neuromuscular junction showing boutons with synapses (white arrowheads), and boutons without them (outlined arrowheads) at larger magnification. The bar scale represents 2 µm. (C) Triple staining done at CT19 and CT7 confirming the presence of synaptic vesicles (Syn, magenta) and synapses (BRP, green) in the presynaptic side of synapses in each bouton (see outlined boutons b1, b2 and b3). Orange dotted lines in b1, b2 and b3 show the places where the analysis of fluorescence intensity was done to confirm the presence of Synorf1 in the proximity of the synapses. Details of the signals obtained are shown for HRP (D), BRP (E) and Syn (F). In (E and F) orange outlines mark selected synapses (S1, S2 and S3) located in boutons marked as b1, b2 and b3. (G) The graph shows the profiles of fluorescence intensity measured for the immunofluorescence corresponding to HRP, BRP and Syn, detailed in (C), (E) and (F). Syn and BRP signals match their location in b1, b2 and b3 and confirm that synaptic vesicles are in the proximity of S1, S2 and S3 synapses. (H) Triple staining confirming the accumulation of glutamate receptors (GluRIIA, magenta) apposed to the accumulation of BRP signals (see outlined boutons b1, b2 and b3). Orange dotted lines in b1, b2 and b3 show the places where the analysis of fluorescence intensity was done to confirm the presence of GluRIIA in the proximity of the synapses. Details of the signals obtained are shown for HRP (I), BRP (J) and GluRIIA (K). In (J and K) orange outlines mark selected synapses (S1, S2 and S3) analyzed in b1, b2 and b3. (L) The graph shows the profiles of fluorescence intensity measured for HRP, BRP and GluRIIA, detailed in (H), (J) and (K). GluRIIA and BRP signals match their location in b1, b2 and b3 and confirm that the accumulations of glutamate receptors correspond with accumulations of BRP in the synapses marked S1, S2 and S3.

Double staining of the MN5 neuromuscular junction showed that not all boutons have synapses (Fig. 3A–B). Triple staining made possible to confirm the presence of synaptic vesicles in the presynaptic side of the synapse (Fig. 3C–G) and glutamate receptors in the postsynaptic side (Fig. 3H–L). Synaptic vesicles were found concentrated in synaptic boutons (Fig. 3C, F, G). Concentrations of glutamate receptors were located in close apposition to the presynaptic marker nc82 as expected (Fig. 3H, L).

**Figure 4:**
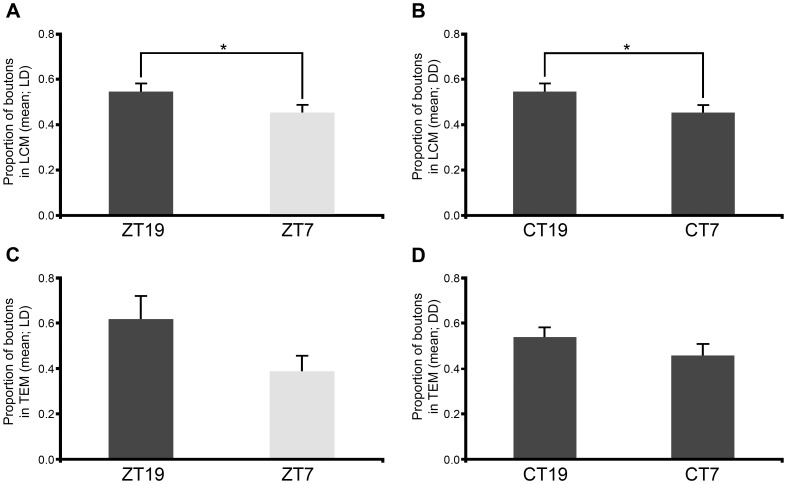
Proportion of synaptic boutons found along the MN5 axon in LD and DD. The graphs show means ± s.e.m in all cases. (A–D) For LCM and TEM, the proportion of boutons per time point was calculated as the number of boutons found in each time point divided by the number of boutons found in the experiment. (A–B) In the LCM samples, the proportion of boutons was higher at ZT19 (0.55±0.04) than ZT7 (0.45±0.03) in LD (A, Mann-Whitney U test, P = 0.03), and higher at CT19 (0.53±0.02) than CT7 (0.47±0.02) in DD (B, Student *t* test, P = 0.04). (C–D) In TEM samples, the proportion of boutons counted in LD and DD showed no significant differences between time points, although the same tendency as in the LCM samples under LD (ZT19, 0.61±0.12 vs. ZT7, 0.39±0.06) and DD (CT19, 0.54±0.08 vs. CT7, 0.46±0.04). Number of flies used in LCM per ZT: ZT19, n = 38; ZT7, n = 43; CT19, n = 33; CT7, n = 37. In TEM: ZT19/ZT7 n = 7 and CT19/CT7 n = 6.

The number of boutons registered with LCM was significantly higher at midnight than midday in flies kept under LD cycles (Fig. 4A and Table 2). The same result was found in flies kept in constant darkness (Fig. 4B and Table 2). The data obtained with TEM was consistent with the LCM findings in LD and DD but did not show statistical differences between time points (Fig. 4C–D and Table 2).

**Table 2:** Summary of the statistical analyses of all parameters studied with LCM and TEM.

Parameter Studied	Method	LD	DD	LD vs. DD
**Proportion of boutons**	LCM	ZT19 vs. ZT7: MW test p = 0.03473	CT19 vs. CT7: t-test p = 0.045062	ZT7 vs. CT7: MW test p = 0.003778
	TEM	ZT19 vs. ZT7: nd	CT19 vs. CT7: nd	ZT7 vs. CT7: nd
**Proportion of boutons** **with synapses**	LCM	ZT19 vs. ZT7: t-test p = 0.0129	CT19 vs. CT7: nd	ZT7 vs. CT7: t-test p = 0.000196
	TEM	ZT19 vs. ZT7: MW test p = 0.03787	CT19 vs. CT7: t-test p = 0.020478	ZT7 vs. CT7: nd
**Proportion of boutons with vs. without synapses**	LCM	ZT19: nd	CT19: nd	ZT7 vs. CT7, without synapses: nd
		ZT7: t-test p = 0.042197	CT7: t-test p = 0.000012	ZT7 vs. CT7, with synapses: nd
		ZT19 vs. ZT7, without synapses: nd	CT19 vs. CT7, without synapses: t-test p = 0.034085	
		ZT19 vs. ZT7, with synapses: nd	CT19 vs. CT7, without synapses: t-test p = 0.034085	
**Distribution of boutons** **with 1, 2 and 3** **or more synapses**	LCM	ZT19 vs. ZT7, 1 synapse: nd	CT19 vs. CT7, 1 synapse: nd	ZT7 vs. CT7, 1 synapse: nd
		ZT19 vs. ZT7, 2 synapses: nd	CT19 vs. CT7, 2 synapses: nd	ZT7 vs. CT7, 2 synapses: t-test p = 0.026557
		ZT19 vs. ZT7, 3 or more synapses: nd	CT19 vs. CT7, 3 or more synapses: nd	ZT7 vs. CT7, 3 or more synapses: nd
		ZT19: Kruskal-Wallis test H(2,N = 114) = 97.0312, p = 0.0000	CT19: Kruskal-Wallis test H(2,N = 99) = 85.4745, p = 0.0000	
		Post hoc MW test:	Post hoc MW test:	
		1 and 2 synapses: p = 2.9×10^−22^	1 and 2 synapses: p = 2.77×10^−19^	
		1 and 3 or more synapses: p = 2.9×10^−22^	1 and 3 or more synapses: p = 2.77×10^−19^	
		2 and 3 or more synapses: p = 4.4×10^−16^	2 and 3 or more synapses: p = 9.71×10^−16^	
		ZT7: Kruskal-Wallis test H(2.N = 129) = 111.9703 p = 0.0000	CT7: Kruskal-Wallis test H(2.N = 111) = 96.7972 p = 0.000	
		Post hoc MW test:	Post hoc MW test:	
		1 and 2 synapses: p = 3.01×10^−25^	1 and 2 synapses: p = 1.15×10^−21^	
		1 and 3 or more synapses: p = 3.0×10^−25^	1 and 3 or more synapses: p = 1.15×10^−21^	
		2 and 3 or more synapses: p = 8.6×10^−21^	2 and 3 or more synapses: p = 5.8×10^−19^	

Light:darkness (LD), constant darkness (DD), transmission electron microscopy (TEM), laser Confocal microscopy (LCM), Zeitgeber time (ZT), circadian time (CT), Mann-Whitney test (MW test), no statistical difference (nd).

**Figure 5:**
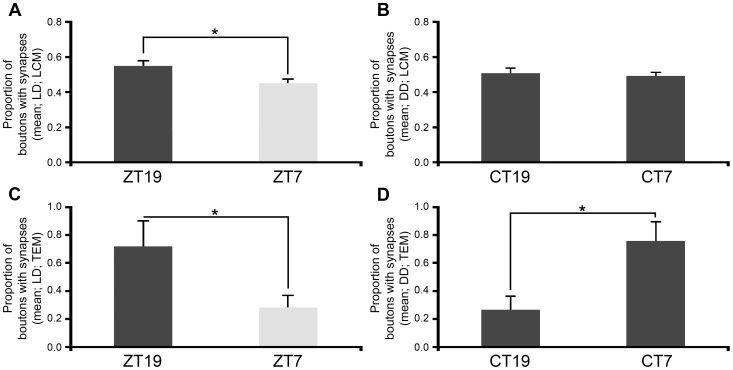
Proportion of boutons with synapses found along the MN5 axon in LD and DD. The graphs represent means ± s.e.m in all cases. (A–D) For LCM and TEM samples, the proportion of boutons with synapses per time point was calculated as the number of boutons with synapses found in each time point divided by the number of boutons with synapses found in the experiment. In LD, a higher proportion of boutons with synapses was found at midnight compared to midday with LCM (A, 0.55±0.03 vs. 0.45±0.02, Student *t* test, P = 0.012) and TEM (C, 0.72±0.2 vs. 0.28±0.08, Mann-Whitney U test, P = 0.037). In DD, a higher proportion of boutons with synapses was found at midday with TEM (D, 0.26±0.1 vs. 0.74±0.15, Student *t* test, P = 0.0205). The comparison of LCM results for LD and DD samples (A vs. B) and TEM results (C vs. D) showed significant differences in the proportion of boutons with synapses between ZT7 and CT7 using LCM (0.44±0.02 vs. 0.56±0.02, Student *t* test, P = 0.0002) but not using TEM (0.34±0.10 vs. 0.66±0.13, Student *t* test, P = 0.06). Number of flies used in LCM per ZT: ZT19, n = 38; ZT7, n = 43; CT19, n = 33; CT7, n = 37. In TEM, ZT19/ZT7 n = 7 and CT19/CT7 n = 6.

After learning that the MN5 has more boutons at midnight, we used LCM and TEM to investigate if this change included a nocturnal increment in the number of synapses. With LCM, we scored the proportion of boutons with synapses. A significantly higher proportion of boutons with synapses were detected at midnight during LD cycles (Fig. 5A and Table 2) but not under DD conditions (Fig. 5B and Table 2). The TEM data confirmed the LCM finding in flies kept under LD cycles (Fig. 5C and Table 2) but detected a reversed relationship in DD conditions (Fig. 5D and Table 2). Although both methods gave a strong indication of the existence of boutons without synapses, we confirmed this feature by serial reconstructions (Figure S1).

In summary, LCM showed that the number of boutons was higher at midnight than midday in LD and DD. Both microscopy methods showed that the proportion of boutons with synapses was also higher at midnight in LD. However, TEM showed that this proportion was higher at midday in DD.

### Synapses were not Detected in a Substantial Proportion of Boutons

**Figure 6:**
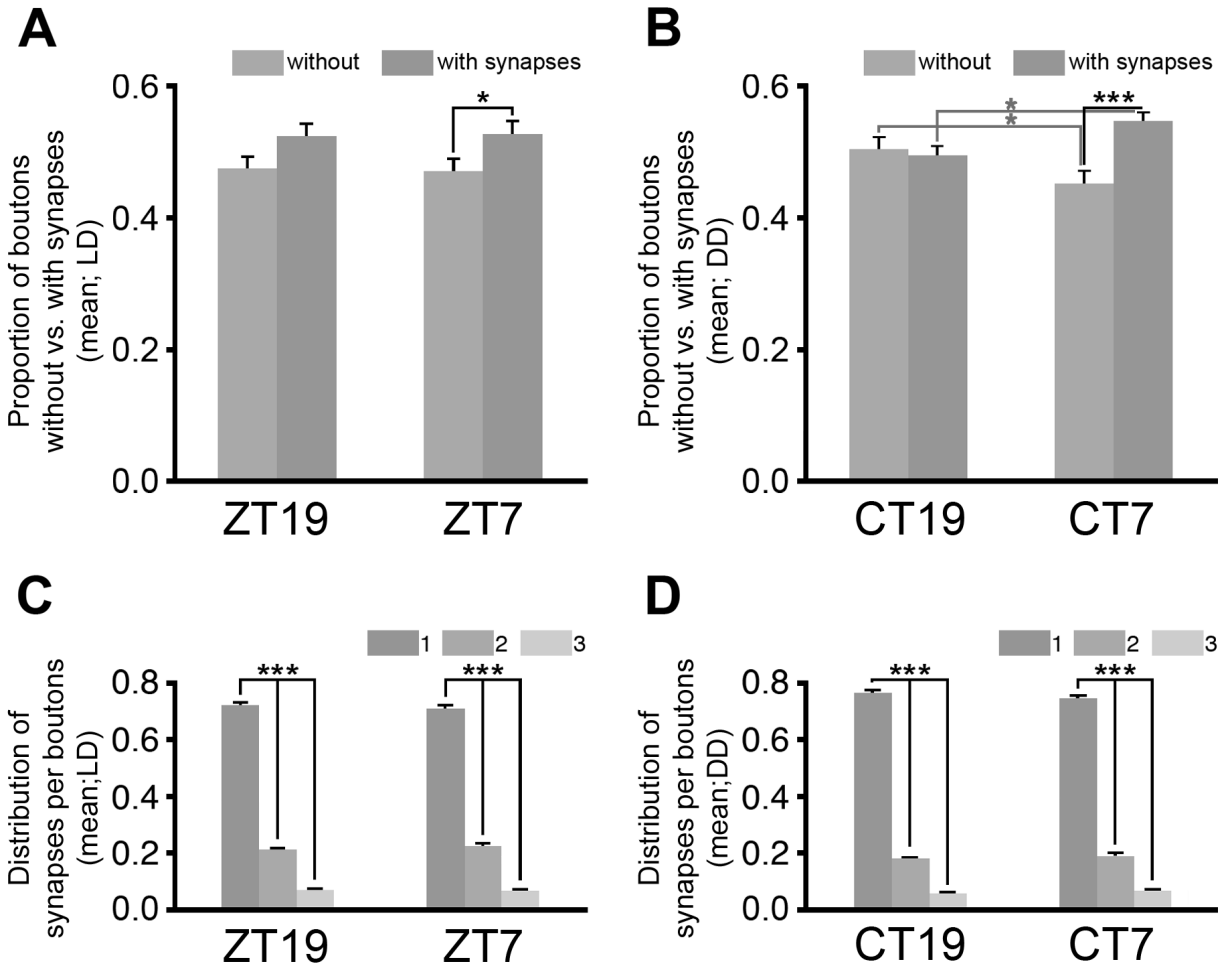
Quantification of boutons with synapses and synapse distribution among boutons by LCM in LD and DD. (A–B) The proportion of boutons with and without synapses (relative to the number of boutons found per time point in each experiment) was around 50∶50 at ZT19 and CT19. However, a larger proportion of boutons with synapses was found at midday under LD and DD conditions (A, LD: ZT7 0.53±0.02 vs. 0.47±0.02, Student *t* test, P = 0.042; B, DD: CT7 0.55±0.01 vs. 0.45±0.01, Student *t* test, P = 0.000012). No significant differences were found between LD and DD. (C–D) The distribution of synapses among boutons was very similar at all time points. (C) In LD, most boutons had one synapse (ZT19: 0.72±0.01, ZT7: 0.71±0.01), few had two (ZT19: 0.21±0.01, ZT7: 0.22±0.01) and even fewer had three or more (ZT19: 0.07±0.01, ZT7: 0.07±0.01) (Kruskal-Wallis test, ZT19: H(2,N = 14) = 97.03, P = 0,000; ZT7: H(2,N = 129) = 111.97, P = 0.000. See Table 2 for Post hoc comparisons). (D) Also in DD most boutons had one synapse (CT19: 0.77±0.01, CT7: 0.75±0.01), few had two (CT19: 0.18±0.01 CT7: 0.19±0.01) and even fewer had three or more (CT19: 0.06±0.005, CT7: 0.06±0.01) (Kruskal-Wallis test, CT19: H(2,N = 99) = 85.47, P = 0,000; CT7: H(2,N = 111) = 96.79, P = 0.000. See Table 2 for Post hoc comparisons). The percentage of boutons with one, two and three or more synapses was approximately 70∶25∶5 at midnight and midday in LD samples (C) and 75∶20∶5 in DD samples (D). The comparison of LD and DD results showed significant differences between ZT7 and CT7 for the subpopulation of boutons with two synapses (Student *t* test, P = 0.026).

The double staining in our LCM samples made possible to study the proportion of boutons with synapses relative to the proportion of boutons without synapses (Fig. 6A–B). Half of the boutons had synapses at ZT19 and CT19 (Fig. 6A–B), but this proportion increased at midday in LD (Fig. 6A) and even more in DD (Fig. 6B).

### The Proportion of Boutons with One, Two or more Synapses is Constant along the Day

The double staining also made possible to analyze the distribution of synapses among boutons, discriminating boutons with one, two and three or more synapses (Fig. 6C–D). In LD, most boutons had one synapse, few had two and even fewer had three or more (Fig. 6C). This distribution was very similar in the DD samples (Fig. 6D). The proportion of boutons with 1, 2 and 3 or more synapses was approximately 70∶25∶5 in LD and 75∶20∶5 in DD, at all time points. The statistical comparison of LD and DD data showed significant differences between ZT7 and CT7 for the subpopulation of boutons with 2 synapses (Student *t* test, P = 0.026). A similar relationship was observed with TEM.

## Discussion

In this study, we present the first evidence for the existence of rhythmic daily changes in synapse numbers in a motor neuron. Contrary to our expectations, in flies kept under LD conditions, the studied flight motor neuron (MN5) had more synapses in the night, when the fly does not fly. Before discussing in detail our principal results we will take up some considerations regarding the methods that we developed to quantify synapses in this particular motor neuron and the reliability of our data.

TEM is certainly the best method to identify synapses regardless of the particular neuron under scrutiny because its optical resolution makes possible for the observer to see individual synapses directly and count them one by one. The level of resolution of LCM is not enough to identify with certainty each individual synapse but this method can be used to count synapses more rapidly than working with TEM, using fluorescently labeled “synaptic markers” which are assumed to give a very good representation of actual synapse numbers [37], [42], [48], [49]. The main disadvantage of TEM is that it demands much longer time per sample than LCM and hence becomes inadequate for the study of the MN5, which has thousands of synapses. Here we found that a combination of TEM and LCM made possible to obtain good replicability (consistency between values of individual flies from each time point at LD or DD) and reproducibility (agreement of the data obtained with different methods and different experiments). In addition, we highlight that our LCM sampling method gave reliable results because the statistical comparison of data obtained from different regions of the muscles indicated that boutons and synapses are distributed remarkably homogeneously across the MN5 target muscles and thus a single image stack per muscle is enough to obtain representative results.

Since boutons are the only portions of the axon where synapses are normally made [11], [14], [69], more boutons during the night would also imply more synapses at night. This would only be true if all boutons contain synapses and if all synapses are uniformly distributed among boutons along the day. Our results show that only about 50% of all boutons have synapses. However, our results also show that synapses are uniformly distributed among boutons along LD and DD cycles. In all samples, the majority of boutons with synapses had only one and the rest had two or more. The maintenance of synapse distribution during the day contrasts with the change in bouton number, suggesting the existence of a homeostatic mechanism regulating synapse distribution among MN5 boutons.

**Figure 7:**
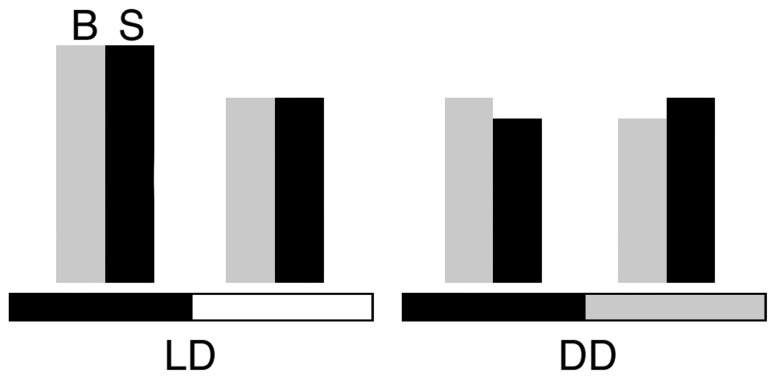
Diagrammatic representation of the daily changes in bouton and synapse numbers found in this study. The bars do not represent exact values and they are only meant to provide a simplified graphic summary of the results detailed in Figs. 4 and 5. In both LD and DD conditions, we found more boutons at midnight than at midday. Synapses, instead, were more abundant at midnight in LD samples and at midday in DD samples. Abbreviations: Boutons (B, gray bars); synapses (S, black bars); light:darkness (LD); constant darkness (DD).

These findings, together with our estimations of the total number of synapses in each sample, strongly indicate that in flies kept in LD conditions the MN5 has more synapses at midnight (Fig. 7). However, we observed the opposite relationship in DD. We do not think that this difference between LD and DD depends mainly on differences in locomotor activity, because it is well established that *Drosophila* flies show a similar pattern of daily locomotion when kept in constant darkness, with more activity during the subjective day [70], [71]. We think instead that this difference indicates the existence of a light-dependent mechanism for synapse assembly/disassembly along the day. It is well documented that light has a positive influence on axonal diameter and on synapse numbers in neurons associated with vision (larger in LD than DD; [19], [30], [32]) as well as on boutons size in the motor neuron studied here (larger in LD than DD; [10]).

The observation of more synapses at midnight than midday in LD samples sounds counterintuitive and as a first explanation we propose the existence of a rhythmic cycle of synapse assembly during the night and degradation during the day. In conformity with this hypothesis, towards the end of the night the fly will start its active phase with a maximum number of synapses, many of which were recently built within boutons of smaller size than those found in the day [10], [25], and loaded with vesicles of larger size than those found in the day [51]. During the day, as the fly flies, the boutons of the MN5 will grow as a result of the continued addition of membrane through the fusion of vesicles used during synaptic activity. At the same time, a proportion of the thousands of synapses made on flight muscles by the MN5 terminal will gradually waste away and be eventually degraded along a global program of ordered synapse disassembly. This would explain the decrease in the number of synapses during the day. The next night, during the phase of rest/sleep, the MN5 will replace these synapses through a wave of synapse assembly which most probably also comprises a dynamic rearrangement of the entire terminal, including the formation of new boutons and their reloading with large vesicles [51]. Our hypothesis is consistent with available data on the time required for the assembly of synapses in *Drosophila*, which takes a few hours in motor terminals [12], [20], [21] and even a few minutes in some brain neurons [19].

This hypothesis can be tested in several ways. An intense wave of synaptogenesis spanning a few hours in the night could leave clear traces in the transcriptome, possibly detectable by, for example, mRNAseq of thoracic tissue samples collected at the right time points. Blocking or reducing the potential of the MN5 to rebuild synapses massively during a short time of the night, using the GAL4-UAS system to drive RNA interference of appropriate genes, would lead to a MN5 with abnormally few and old synapses the next morning. Electrophysiological studies could address whether the day after such an experiment the MN5 will function with a set of relatively “aged” synapses.

In conclusion, in flies kept under LD cycles the MN5 has a daily rhythm of bouton formation/elimination and synapse assembly/disassembly. New boutons and synapses are formed in the night, when the fly is resting, and some boutons and synapses are eliminated during the day, when the fly is active.

## Supporting Information

Figure S1 **TEM serial reconstruction of a bouton without synapse.** The glia is depicted in green. Scale bar represents 200 nm.
(TIF)

Table S1 **Total number of boutons and boutons with synapses found with TEM in each sample/fly.**
(XLS)

Table S2 **Total number of boutons, boutons with synapses and boutons without synapses found in each sample with LCM.**
(XLS)

Table S3 **Measurements used for the Analysis of Reliability of the sampling method.**
(DOC)
