# Supplementary figures and images for: Rhythmic Changes in Synapse Numbers in Drosophila melanogaster Motor Terminals
Source: PLoS One. 2013 Jun 28;8(6):e67161. doi: 10.1371/journal.pone.0067161 (PMC3695982; doi:10.1371/journal.pone.0067161)

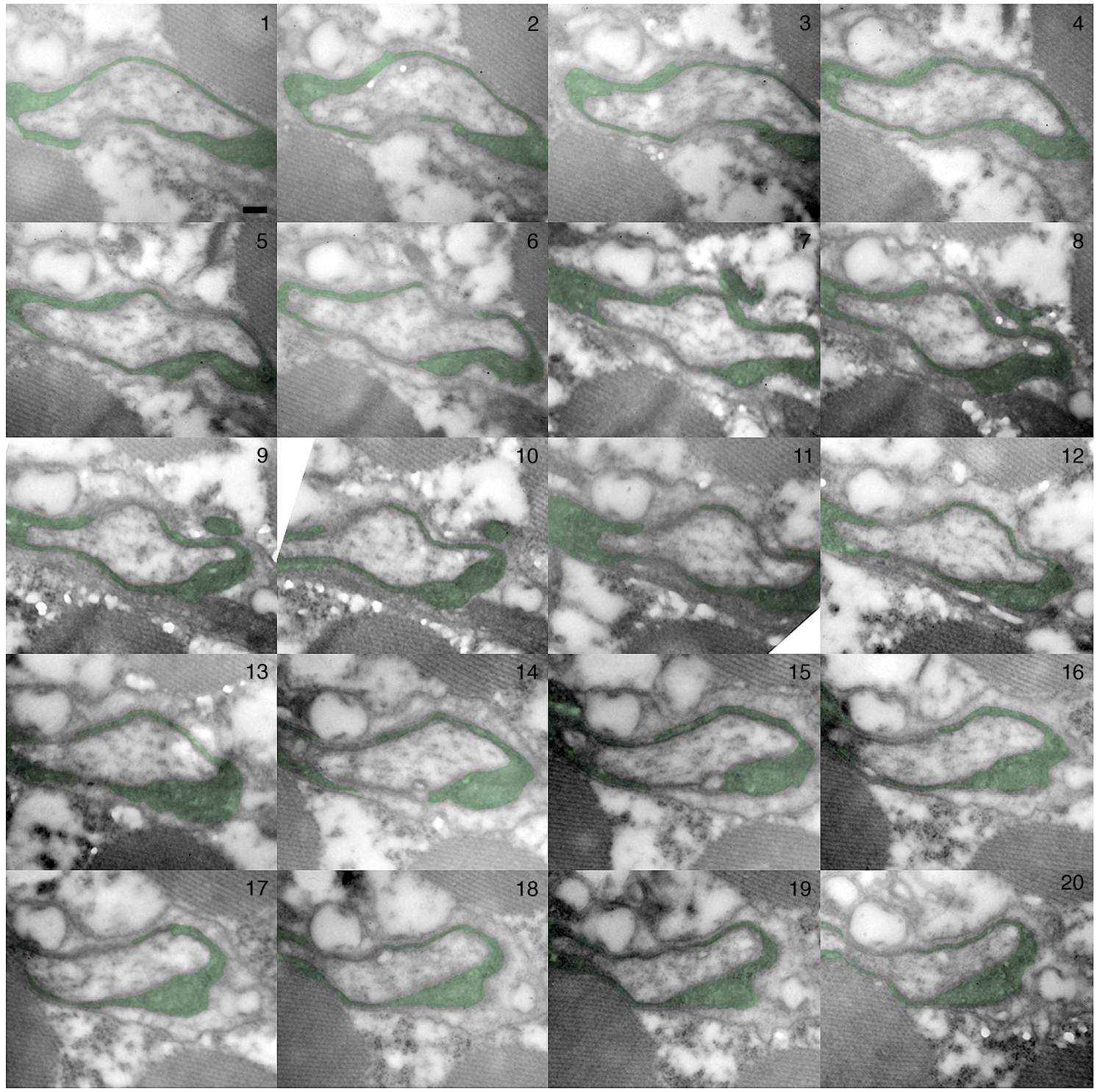

Supplement: Figure S1 — TEM serial reconstruction of a bouton without synapse. The glia is depicted in green. Scale bar represents 200 nm. (TIF) [file pone.0067161.s001.tif]
