# Supplementary material for: Rhythmic Changes in Synapse Numbers in Drosophila melanogaster Motor Terminals
Source: PLoS One. 2013 Jun 28;8(6):e67161. doi: 10.1371/journal.pone.0067161 (PMC3695982; doi:10.1371/journal.pone.0067161)
Supplement: Table S3 — Measurements used for the Analysis of Reliability of the sampling method. (DOC) [file pone.0067161.s004.doc]

Table S3. Measurements for the Analysis of Reliability of the sampling method.

| **Muscle region** | **Total boutons** | **Boutons with synapses** | **Boutons without synapses** | **Total synapses** |
| --- | --- | --- | --- | --- |
| AS IFM5 | 209 | 112 | 97 | 142 |
|  | 79 | 36 | 43 | 49 |
|  | 180 | 126 | 54 | 160 |
|  | 119 | 74 | 45 | 103 |
| Mean | 146.8 ± 29.4 | 87 ± 20.2 | 59.8 ± 12.6 | 113.5 ± 24.6 |
| AS IFM6 | 118 | 71 | 47 | 103 |
|  | 170 | 102 | 68 | 132 |
|  | 137 | 92 | 45 | 120 |
|  | 119 | 77 | 42 | 102 |
| Mean | 136 ± 12.1 | 85.5 ± 7.1 | 50.5 ± 5.9 | 114.3 ± 7.2 |
| MS IFM5 | 137 | 86 | 51 | 114 |
|  | 162 | 101 | 61 | 137 |
|  | 148 | 91 | 57 | 124 |
|  | 143 | 92 | 51 | 124 |
| Mean | 147.5 ± 5.3 | 92.5 ± 3.1 | 55 ± 2.4 | 124.8 ± 4.7 |
| MS IFM6 | 247 | 121 | 126 | 155 |
|  | 133 | 84 | 49 | 115 |
|  | 163 | 105 | 58 | 135 |
|  | 183 | 128 | 55 | 177 |
| Mean | 181.5 ± 24.1 | 109.5 ± 9.8 | 72 ± 18.1 | 145.5 ± 13.3 |
| PS IFM5 | 108 | 62 | 46 | 78 |
|  | 124 | 81 | 43 | 107 |
|  | 234 | 152 | 82 | 200 |
|  | 103 | 71 | 32 | 99 |
| Mean | 142.3 ± 30.9 | 91.5 ± 20.5 | 50.8 ± 10.8 | 121 ± 27.0 |
| PS IFM6 | 169 | 101 | 68 | 130 |
|  | 109 | 66 | 43 | 91 |
|  | 198 | 130 | 68 | 173 |
|  | 106 | 73 | 33 | 93 |
| Mean | 145.5 ± 22.7 | 92.5 ± 14.6 | 53 ± 8.9 | 121.8 ± 19.3 |
